# Judicial breakfast as an external factor in judicial decision making in courts

**DOI:** 10.12688/f1000research.126482.1

**Published:** 2023-01-04

**Authors:** Manotar Tampubolon, Tomson Situmeang, Paltiada Saragih

**Affiliations:** 1 Faculty of Law, Universitas Kristen Indonesia, Jakarta, 13630, Indonesia

**Keywords:** court, extraneous factors, formalism and realism, legal realism, judicial decision making, judicial officers, judicial rules and laws, judicial officers.

## Abstract

The objective of this article is to establish whether the judges depend on relevant facts, judicial rules, and the law when making their judgments or they use extraneous factors such as what a judge eats, personal ideology, beliefs, or the cultural and political environment. The discourse between the two sides is incomplete without exploring the grand theories: formalism and realism. The antimony between the two theories resulted in theoretical analysis and empirical research. The realism challenged the existing logical reasoning and legal rules that judges use in making their judgment as they contend that judges applying rules and law in their decision-making process are irrational and mechanical. Formalism insists on using the judicial rules and the law in making decisions as opposed to extraneous factors, which realists contend should be the basis for decision making with laws and rules only to support the findings. The continental legal theory holds that legal realism is a hard-nosed, down-to-earth, and practical school of thought that is opposed to mechanical and scientific theories. The scholarly analysis of the judicial decision-making process brings into focus the conduct of judicial officers and whether they base their reasoning on extrajudicial issues. However, the discussion should avoid denigrating into an attack on the personality of judges as it undermines the rule of law.

## Introduction

The adage that judicial decisions by judges depend on what they ate in their breakfast presents a discourse on whether outcomes of legal cases rely heavily on facts and laws alone. Legal formalism refers to the systematic, logical, and purposeful application of facts and arguments to situations by judicial officers ( Aiken & Shalleck, 2016). The alternative claim is that legal realist movements in the 20th century promoted the judicial idea that decisions are grounded in the logical application of the law. It was observed that the life of the law is premised on experience rather than the law. The Supreme Court Judge, Oliver Holmes argued that the balanced application of the law is dependent on the political, psychological, and social factors rather than only legal reasoning and underpinnings ( Aletras *et al.*, 2016; Auerhahn *et al.*, 2017; and Bator, 2020). The old trope that judges’ decision-making process is dependent on what they ate presumes that law is subject to imperfections, biases, and foibles that affect human cognitive function. Indeed, people would appreciate it if judges made their ruling based on the written laws and rational decisions; however, the reality is that their decision is influenced by irrelevant factors such as their breakfast and mood.

Existing research shows that making repeated decisions and judgment often depletes the mental resources and executive functions of the individual, which influences their decision-making process ( Bonica & Sen, 2021; Boyd, 2016; and Bozorgmehr & Naseri, 2020). Sequential decisions between consumer goods affect the intuitive decisions of humans and lessen the tolerance to pain, which makes them tend to simplify decisions. Social science assumes that the decisions that human beings make are dependent on rationality, are unemotional and logical. However, the neoclassical price theory hypothesizes that consumers and producers are rational actors but in law a reasonable person is a cousin to economic actors ( Burns, Dioso-Villa & Rathus, 2017). The dominant decision-making process in the judicial industry is an extension of the rational choice theory where the judicial officers are rational actors that base their actions on previous decisions, facts, constitution, and statutes. The problem with the theory is that it only accounts for a portion of decision-making processes since, from a realist perspective, non-doctrinal considerations were disregarded even though they have an impact on judicial judgments' outcomes. Cognitive scientists and behavioral psychologists have explored how the brain operates, which brings the foundation for examining the actions of judges. Studies show a surprising correlation between emotional and cognitive health and the state of the digestive system, which highlights the importance of judges making a decision when they take breakfast ( Chen, 2016). To understand how judicial decision-making is performed, understanding how the mind operates is critical in validifying or refuting the claims that judges make a decision depending on factors other than the law and facts. Establishing whether there are existing choices, competing priorities, and decisions that judges make, as well as their perceptions on issues, is critical.

## Methods for assessing the decision-making process

The adage that judges make a decision based on what they consume presents a discourse on factors that judges use when making judgments in courts. The realists contend that the courts are influenced by extralegal factors when making judgments while the formalists contend that judges only rely on facts, laws, and legal rules ( Chen, Moskowitz & Shue, 2016). The realists assert that judges do rely on precedence, and are influenced by policy perspectives, and personal feelings, which the formalists disagree on. They argue that judges are not scientists that make a mechanical decision in adherence to rules rather than reality ( Chen, 2019). On the other hand, the formalists contend that giving judges free will to make judgments based on their personal opinion, beliefs, and values departs from constitutionalism. To settle the differences, testing for factors affecting the judicial decision-making process requires an experiment. Following DLA theory, the study will seek to establish whether judicial decisions only consider facts, laws, and legal rules or are influenced by extraneous factors.

For realists, the use of scientific methodology such as experimental research designs is essential in answering the question. Hypothetically, they can conduct an experiment that involves two groups, an experimental group that tests the independent variable and a control group that measures the dependent variable. On the one hand, the experimental group should be made of experienced legal practitioners who have handled many cases. These lawyers are provided with a large sample of cases, an assembling of facts, and asked to predict the outcome of the case. They should use the facts and the number of cases to predict the reasoning of the court. In this group, the participants will not be provided with information such as names and personalities of judges, judicial locale, parties in the case, prevailing cultural and social norms, and emotional appeals that lawyers presented during the hearing of a case. In essence, the participants will be provided with formal legal rules and facts devoid of other extraneous factors such as what the judges ate or how tired they were. On the other hand, the control group will be provided with facts about a case and asked to make predictions about the court reasoning and outcome of the case. They will be denied formal rules and asked to predict the ruling; possibly, by tossing a coin. The control group should be made of a large sample size capable of producing high confidence levels of about 50%.

The research question for the study would be whether the lawyers could predict the court ruling correctly while using the legal rules and laws to make the predictions compared to the control group. Based on the reasoning of realists, the answer to the question would be largely clear and favorable to their assertion that it is difficult to predict a court decision when only the law and legal rules are presented. Realists argue that scientific theory adopted by formalists is wrong or incomplete because it can only explain events in the past, but cannot predict future events. The realists would agree that it is possible to make predictions when a scientific theory that uses legal rules and law to make judgments is adopted together with policy principles, the personality of a judge, attributes of litigants, judicial ideology, cultural elements during the hearing, and the emotional elements present in the case.

The findings of the study can be confirmed by contemporary empirical studies. Research conducted by Posner notes that the outcome of a Supreme Court case in the US can be predicted when variables are assessed, which includes no legal doctrine as opposed to a set of experienced constitutional lawyers with facts and an understanding of legal rules. The main objective of the realists is to develop a theory of judging by establishing the factors that determine judicial decision-making. Examining the decision-making process in the judiciary until one can confidently predict judgments is at the center of realists' studies. Though the realists argue against the assertion that law is a legal science, their proposition is best explained using a scientific inquiry.

## Literature review

### Constraints

The constitution allows the judge discretion to make decisions as they are permitted to develop findings of facts, design remedies for violation of the law, interpret the language of the constitution, regulations, and statutes, and grant injunction relief. As well, the judges are allowed to establish whether the government officials in the executive arm have abused their discretion ( Cserne, 2020; Dagan, Kreitner & Kricheli-Katz, 2018). However, the reality is that the judges are constrained on how they exercise these powers as they can't act on their desire. First, the judges are constrained by their self-respect as they consider their rulings examined and assessed by many people. When judges make a ruling, they consider how practitioners consider their reasoning, which should be based on solid grounds, facts, and reasonable suppositions ( Diamond, 2019). Law practitioners expect judges to make decisions and support them with convincing facts. When they choose to avoid following a principle for them to reach an outcome, it is difficult to argue that one is following the law when making a ruling ( Gordon, 2020). Whether one thinks that the judgment is wrong or right, their moral compass and self-respect constrained them into making decisions that are right regardless of their desires.

Elliott (2016) adds that the judges are constrained by their colleagues as their decisions are reviewed by the judges in the upper courts. As such, they are forced to make rulings that are convincing, accurate, and adhere to the law. It is integral that they decide to use convincing facts as they have to persuade the upper court that their ruling was correct and reasoning valid. In hearing an appeal, the oversights and errors that a judge in the lower courts makes are often paraded which can be embarrassing. As such, the judges consider the opinions and stances of their colleagues to avoid embarrassment during the hearing of an appeal ( Hamer & Edmond, 2016). Although the judges can cut corners with their judgment, their actions often catch up with them as their opinions can be subjected to scrutiny by the Supreme Court. There are many cases with no time and resources to catch all the errors whether they were unintentional or deliberate. However, it is difficult for judges to avoid using the legal rules in courtesy of results consistently. Avoiding adhering to the rules and regulations makes a judge a target of their colleagues ( Gravett, 2017). During the appeal, judges that decide by breaking the rules face a reversal in Supreme Court and are vulnerable to *en banc* calls.

Also, the judges are constrained by the political system that checks the judicial excesses, though the factor is often discounted. The political system and process rarely respond to the specific issue in a court ruling, but it does when the contentions affect a significant group. For instance, the Supreme Court experienced intense pressure from Congress on the Civil Right Act of 1991, which they believed was misread ( Eliot, 2021; Fine *et al.*, 2017; and Geerling *et al.*, 2017). As such, they decisively and swiftly moved to overrule the decision of the Supreme Court by statutes when they felt that the court erred in its judgment and application of the law. The political pressure constraints the courts in blunter ways; for instance, FDR planned a proposal to clip the jurisdiction of the federal courts on the sensitive matter and pack the Supreme Court ( Glöckner, 2016). Also, the voters voted to remove all the three justices in the Supreme Court in California. The voters were persuaded politically that the justices were incompetent and unfair or using their position to engraft political changes in the country. It shows that justices in the Supreme Court are aware that sensitive political matters require deliberate analysis and assessment since it implies their position. The politicians can incite the public against the court's lading to their removal; hence, become sensitive in their reasoning ( Harris & Sen, 2018). There is a premise though unspoken that there are less or more objective principles that the law operates, rules that determine the reasoning as established in a court ruling. The judges may not follow these principles; however, they understand their impact on their judgments.

Principles like language are integral for judges to use when making their rulings. The choice of words in a statement can have diverse interpretations; thus, judges are inclined to use the appropriate words that defend their reasoning. There are words with immutable and single meanings while others are debatable ( Heumann *et al.*, 2019; Hou & Wang, 2020). While reading the statutes, contracts, and regulations in the constitution brings constraints to a judge when deciding on cases. The language used presents an outer boundary that judges use to interpret and apply in their reasoning though there are precise lines with varied meanings. Though fairly strict, the language used is generous to judges though constraints are evident, for example, Article II, Section I, and Clause 5 in the constitution state that persons eligible for the office of the President in the US shall only be a person born and be US citizen ( Zygmunt, 2020). The wording in the constitution gives no room or little space for interpretation. The language used offers meaningful and firm constraints on who is eligible for office as a US president though some can debate that an individual born in the US by one parent from a foreign country is ineligible to contest for the office of the predicant in the US. The case in point is that of Obama who faced intense opposition from individuals who believe that he was a foreigner as one of his parents was a Kenyan. At the same time, others can argue that a person born to both American citizens in a foreign nation be considered a citizen by naturalization. Also, another case in point is the Fourth Amendment which proscribes arbitrary and unreasonable seizures and searches ( Jiménez, 2021). However, the term ‘reasonable’ raises concern as it is debatable; however, the rulings of courts aren't made in a vacuum. The interpretations of the term ‘reasonable’ reasonable are shared on the notions of personal autonomy and individual privacy. In essence, regardless of the judge's reasoning, warrantless searches on a specific block are not considered reasonable ( Jiménez, 2021). The marginal cases present challenges in establishing line-drawing problems, which affirms that language poses constraints on the manner in which courts interpret cases.

Moreover, the judgement presents constraints on how the courts make their rulings. The judges are forced to consider precedence rather than ignore them when making their judgments. They should avoid distinguishing it based on a trivial and unsubstantiated basis. It is difficult to find judges writing their ruling without citing precedents, which offers a constraint on how judges decide ( Winter, 2020). Precedence in courts, just like language, provides an opportunity for judges to make their determination; however, dishonesty and judgments are clear when using or burying precedence. Lawyers who fail to provide citations of their arguments with precedence often incense the judges. The lawyers are expected to carefully cite their precedence as an authority for their arguments and properly state the holding cases in a principled manner ( Leibovitch, 2016). Lawyers find themselves in a problem when they find two lines of authority are cited within the same subject that provides different lines of discussion. As such, it becomes challenging for lawyers to advise their clients on the rule of law and how to conduct their affairs depending on the judges listening to their case.

Pichlak (2020) says that every individual has interests, biases, instincts, and learning that constrain their judgments. Though integral issues, these factors are often ignored in the legal profession. Like all humans, the judges do rely on their instincts as facts and evidence can be masked which affects one’s ability to perceive issues with objectivity ( Leiter, 2020). The approach can make a difference when the judges consider their gut feeling or hunches in a case. The facts can be misleading which can lead to the wrong decision by judges; however, when they consider their hunch, they can unmask lies concealed as truth. Nonetheless, it is critical that one is skeptical of their instincts and doubts their leanings as it can result in a wrong conclusion. Establishing the difference between what a person thinks and the decision that they make it difficult to discern ( Mańko, 2021). Consequently, it is challenging for interested parties to predict the outcome of a case when they understand the leanings of a judge. However, as realists contend, it is easy to predict an outcome of a case when a person has information about the judge as they are likely to take sides on what they believe. It provides an opportunity for lawyers to fight these impulses rather than yield to them.

### Extraneous factors in judicial decision making

Judging is a self-indulgent job with serious ramifications and hazards. The judges are expected to constantly avoid their bias, leaning, persuasion, and instincts when deciding on cases. The judges hold a special role in society as arbiters of cases and custodians of the constitution; hence, their egos and leanings should be abandoned in courts. When trusted, the judges can make good rulings that are acceptable to the public ( Weinshall, Sommer & Ritov, 2017). They are called to make judgments on cases and issues that are unpopular to the public; hence, expected to be impartial and reasonable in their rulings. For instance, they are expected to strike out legislation on a matter of public interest, release convicted criminals or terrorists, or allow protests by people perceived as an offending society. The courts expect people to follow their rulings even when they are against their expectations ( Menga, 2018). The basis for their argument is that the constitution was created to cause unpopular sacrifices that protect everyone in the society, even when they are a minority.

Western practices and norms place severe restrictions on the conduct of a judicial officer, which is expected to be fair, rational, and acceptable. Their actions and decisions should be premised on the main tenet, which is the rule of law that should be applied to the same situations and individuals similarly. The rule of law distinguishes judicial decisions since it is premised on law rather than on power. As an arbiter, a judge is expected to be a disinterested arbiter since it promotes corrective justice ( Mertz, 2016). The legal profession has internalized the requirement of disinterest as judicial officers should behave in an appearance of approximate pure neutrality. Even when judges are confronted with emotional elements that are challenging and messy, they are sworn to make decisions and determine issues based on customs and laws of the land rather than their private judgment. They are restricted to basing their arguments on popular opinion, pressure from other arms of government, and personal convictions ( Mitchell, 2019). Considering the above discussion, judicial conduct premised on personal preferences, popular whims, and political pressure is considered illegitimate.

In legal education, the practitioners are taught the value of the importance of observing rule of law. The focus on the current legal rules is emphasized to create precedence with policy consideration ignored unless forced through the presence of precedent ( Mindus, 2021; Mocan, 2020; and Owens, 2016). Public policy is considered a cost-effective argument as opposed to policy goals such as justice and equality. Unsurprisingly, the judges are hesitant to consider or admit that anything else apart from pure legal stance influences their decisions and rulings. In many instances, the judges are often attacked for judicial activism, which means that they are influenced by ulterior motives other than the law. The judges often note that they are often forced to take counter-majoritarian positions in support of their rulings ( Pasquale & Cashwell, 2018). As well, there exists cynicism on whether the legal decisions reflect the reality as the degree of human perfection, just like all customs are probabilities with lived experience. The judges are surrounded by friends, family, life, expectations, and connections that often shape their actions and behaviors when they are off the bench ( Pichlak, 2020). A judicial officer with particular outlooks, connections, and positions in society tends to develop stances inclined toward political and social influence. It is difficult to ascertain whether the judges are immune to influence from their religion, political stance, and economic realities when they make decisions and rulings.

In the long term, an overly rigid judge who fashions adherence to rule of law would be counterproductive to their rightfulness in the courts. The interpretation of Aristotle's idea of justice presents a unique perspective since it emphasized lack of empathy. The decisions of individuals in different contexts affirm that people don't rely strictly on rationality in making choices and judgments as they are often influenced by irrelevant facts such as the presence of random anchors or changes in presentations ( Priel, 2020). In the judicial realm, the legal cases should be decided based on relevant facts and law only with minimal extraneous factors. In principle, the decisions of a judge should be devoid of influences such as whether a judge is hungry, exhausted, or the manner in which a case is presented. Still, studies have shown that the judges present biases and fallacies to individuals when they decide on a matter in courts ( Quintanilla, Alle & Hirt, 2017). In essence, the decisions of judges involve intuitive and constructive elements rather than those premised on pure rational calculations, which makes their ruling malleable to irrelevant facts.

The judges play a critical role in protecting and progressing constitution systems and since they are appointed by political actors rather than elected, they experience a democratic deficit. It means that they lack the mandate and authority to make decisions based on their personal policy preferences when making judgments on disputes ( Richards, 2016). Though people expect that judges should serve as impartial arbiters, and make a decision based on law and constitution rather than personal preferences, they are constrained by other issues. The basic assumption of the conventional legal reasoning is that judicial discretion is limited, which means that the decisions of judges are constrained by the precedence, text, and reasoned inquiry on the selected drafting officials and the intent of the constitution. Studies that explored the impartiality of judicial decisions concluded that the behavior of judges often decides in favor of judgments that are inconsistent with their policy stance ( Ranieri, 2019; Schultz & Kovacs, 2016; Singh, 2018). The focus is on inter-agreement and dissident, which shows that the decisions of judges in some instances border on extra-legal elements. Critics of Supreme Court judgments contend that the institution is political as the justices make decisions that border on and reflect their personal and political values ( Smejkalová, 2020). The court is provided with the powers to act on governance issues, which makes the justices at the Supreme Court targets of the impeachment process by Congress. When judges are perceived as partisan, the critics argue that they are guided by political influence and policy preferences ( Song, 2019). Besides, the media coverage of the cases in the Supreme Court fuels the perception that political influence plays a role in the determination of cases. Though the press analysis of a court ruling does little to placate the description of judgments as politically influenced, the narrative satisfies political influence.

### Theoretical framework

Legal literature holds that the conventional perspective of legal judgments is made logically and mechanically from the official legal materials such as court cases and statutes, which has often been challenged by legal realism. Legal realism contends that the legal judgments and doctrines are more malleable, less causal of judicial outcomes, and less determinate as opposed to the conventional view that the constraints propose ( Spence, 2016). Legal realism believes that extraneous factors and official legal materials influence court rulings. The extraneous factors such as judgment bias, policy and ideological preference of a judge, and intuition guides the judges when making decisions. Legal realism holds the old trope that “justice is what the judges ate for breakfast” ( Tejani, 2016). Analysis of the legal decision-making can be explained using theories such as explained by formalism and realism theory, DLA, democratic and Hutcheson Theories ( Tejani, 2016).

### Danziger, Levav and Avnaim-Pesso Theory (DLA)

Judicial decisions are influenced by extraneous factors as opposed to facts and law. The Danziger, Levav, and Avnaim-Pesso (DLA) theory contends that deciding on several cases simultaneously influences the legal outcomes of a judge. The theory premised its finding on the study of 1,112 legal rulings in Israel conducted by the parole board, which handles most of the parole cases in the country ( Teichman & Zamir, 2021). They assessed the order of case presentations where they learned that the board handles three cases daily, which are separated by breakfast and lunch breaks. Comparing the first and the last ruling, the study found that favorable outcome drops by 60%. According to the research, the likelihood of a good result decreases from 60% in a first option to zero in a last one ( Wei, 2021). As such, the theory established that the judges are influenced by extraneous factors, which they speculate was attributable to depletion of mental cognition. They argued that when the judges make decisions, they become hungry, exhausted, and mentally drained, which forces them to use an effortless and simple strategy to retain the status quo ( Troop, 2018). As a result, the number of rejected cases increases since the judges are influenced by their hunger and exhaustion leading to the “irrational hungry judge effect” ( Troop, 2018).

Judges face challenging tasks when making decisions with the explosion of fact and indeterminacy of the law. They are floating in the sea of documents, oral courtroom testimony, affidavits, deposition transcripts, and expert opinion that presents a daunting task when deciding cases without reducing and simplifying factual complexity ( Bonica & Sen, 2021; Boyd, 2016). The use of case management is adopted by judges to manage the increasing complexity and crushing caseloads; however, consensus holds that law is indeterminate. Among the legal circles, there is a challenge to determine how the judges manage to make informed choices when faced with an explosion of facts. It is difficult to perceive judges as scientists that make decisions like technicians who make choices based on deductive logic ( Bozorgmehr & Naseri, 2020). The traditional view is that judicial decision-making should be made based on the application of law and legal rules while others contend that they should make decisions to realize the specific aim or end economic efficiency. Realism argues that judicial decision-making is circumscribed by political power and reasons rather than the law and legal rules ( Burns, Dioso-Villa & Rathus, 2017). To understand judicial decision-making, realists contend that deconstructing the reasoning of judges helps to determine hidden meaning and presuppositions such as gender, race, and class that guides it.

### Hunch Theory

Hunch theory by Judge Hutcheson on judicial decision-making offers an alternative explanation of the approach embraced by judges when making decisions. The theory acknowledges the importance of judges when making decisions in society and the legal indeterminacy. Hutcheson contends that it is difficult to reduce law into logic as judges are not technicians to determine cases mechanically ( Carvacho, Droppelmann & Mateo, 2022). However, the judge notes in the court that judicial decision-making cannot be reduced to politics and made an issue of technical reasoning. The decisions should be premised on an empirical and pragmatic approach, which means that judges are allowed to feel and intuit on decisions that they make. When making decisions, judges must consider all the materials presented and use their hunch or intuition to guide their determination ( Chen, 2016). The imaginations of a judge play a role in lifting their mind beyond the conflicting and constricting facts, as well as precedence that can impede just and fair judgments. Through this method, a judge's intellect can be exposed to the full extent of his/her experience, enabling resolution of cases ( Chen, 2019). In essence, the hunch theory contends that judges should focus on factual complexity in the presented materials to establish the legal significance without containing and reducing the power of technical reasons.

Hunch's theory accounts for the judicial decision-making that is perceived as similar and familiar to indirect and imprecise approaches to deciding on issues that affect individuals in their daily life. The use of terms such as intuition and hunch mean that there is potential arbitrariness and rudeness in the manner that judges make decisions ( Chen, Moskowitz & Shue, 2016). The potential challenge with the assumption is to determine whether the judges decide by guessing. It is difficult to presume that judges made a decision based on hunches alone; hence, the case requires epistemological justifications. Anything would be permitted if the hunch theory is validated arguing that judges make decisions by guessing. The adoption of the hunch theory is characterized as advocating for the trope “law is a matter of what a judge had for breakfast” ( Cserne, 2020). According to William James’ pragmatism ( Olin, 2020), the judges' decisions based on hunches save the arbitrariness, which offers compelling epistemological justification.

The hunch theory presents a practical explanation and solution for the indeterminacy of the law and the explosion of facts. As such, judges should apply their hunches until they can realize pragmatic conditions established for the justification. These conditions fail to offer a false sense of judicial constraints and legal certainty in the same way legal formalism provides ( Dagan, Kreitner & Kricheli-Katz, 2018). Nonetheless, the requirement that judges experience the idiosyncratic sense of certainty on the rulings that they make and test pragmatically on the impact of their hunches pronouncements offers a disciplining effect on the judges as they depend on subjective hunches. Though legal realism is founded on pragmatism, hunch theory contradicts the main tenets of legal realism ( Diamond, 2019). The legal realists contend that legal interpretation is premised on law rather than science, which characterizes them as rule skeptics. Legal realism has adopted a scientific approach and narrowed the perception of experience in attempts to reform the law by making it more scientific and rational.

### Democratic Theory

The democratic theory contends that the framers of the US constitution designed measures that insulated the judicial actors from politics, which helps them to make judgments and pronouncements based on their expert interpretation of the law. Consequently, the judges are given the freeway to make judgments that are consistent with their policy preferences. Segal and Spaeth contend that the voting behaviors in Supreme Court by judges highlight the attitudinal responses triggered by differential case stimuli ( Gordon, 2020). The choice of judges is influenced by their policy preferences as established in the litigation. Supreme Court Judges were not democratically responsible for their rulings since they are employed by the institution for rest of their lives ( Eliot, 2021). As a result, they make choices that incline them to show their policy preferences. The argumentative nature of the legal system gives room for judges to make decisions premised on the arguments that are compelling to the competing groups. The law permits judges to make decisions without constraining them, which makes them make justifications consistent with their choices after rendering their decisions ( Fine *et al.*, 2017). In essence, it shows that they are allowed to take policy preferences rather than base their judgments on legal authority.

### Formalism and Realism Theory

The legal formalism theory contends that there is a pyramid of rules where first principles, middle level, and specific rules guide how judicial decisions are made. It means that when judges are making their ruling, they consider the law and determine the rule that provides the opportunity to make a correct determination ( Geerling *et al.*, 2017). The formalism theory contends that for every case, a judge should explore the differences and similarities of previous cases that are classified in taxonomy, which makes the ruling accurate. In law, the basic principles are discerned by induction from rule of law and cases that were premised on principles and decided on from rules ( Glöckner, 2016). The critics of legal formalism contend that the life of the law is based on experience rather than logic, which means that the ideologies are not decided by concrete cases. The principles of law are not derived from a watchful analysis of principles and rules, which means that there is a fuzzy overlap where a judge creates a distinction as opposed to discovering it ( Gravett, 2017). The differences are often influenced by the sense and perception of a judge, which means that it is often illogical and arbitrary.

The legal formality theory contends that legal rules and fundamental principles are critical as they offer guidance to judges though insufficient in determining the outcome of cases. The sense of inevitability and certainty as expressed in judicial opinion was considered unjustified by critics ( Hamer & Edmond, 2016). However, the legal landscape responded to the failures of formalism as it became dense with intermediate cases. Although two cases can seem different and the distinction is easy to establish, a cluster of cases makes it challenging to trade the difficulty. As such, the determination of cases is based on the preponderance of feeling as opposed to the articulation of reason, rules, and principles ( Harris & Sen, 2018). Though the courts are avoiding the idealistic theory that bred formalism, the analytical methods are present. For instance, there is a robust classification of cases as procedural law, property, torts, and contracts versus substantive law. The tenets of formalism theory form the basis of many judicial opinions, which means that the rulings are premised on rigorous analysis of acceptable law and facts ( Heumann *et al.*, 2019; Hou & Wang, 2020). The judge discovers the governing principles to help in making the correct decision. There is no room for errors and doubts as the neo-formalist jurisprudence is characterized by self-reported involvement of restraint, singular correctness, and high confidence.

Conversely, legal realism theory contends that laws in a country are derived from the existing public policy and social interests, which means that these considerations rather than abstract rules form the basis for the decision of a judge. As aforementioned, the legal realism movement started when law practitioners challenged the existing view that judges are rational arbiters who rely on law and legal rules in making their determinations. When making a decision, judges are expected to determine cases by extension exploring their policy perspectives, principles, and mind before turning to law and legal rules ( Jiménez, 2021). Scholars consider legal realism as a controversial theory that, though influential, is often misunderstood. It is a school of thought that distorts and homogenizes court decisions rather than simplifying them. The premise of legal realism is that a judge should develop a preferred outcome for a case before they explore the existing law and legal rules ( Leibovitch, 2016). The preferences are based on a non-legal basis such as the public policy preferences, the conception of justice, ideology, the personality of a judge, and the attributes of litigating parties like a poor plaintiff, government, or racial group. After making preferences, the judges look for justification for their choices using the legal rules and law ( Leiter, 2020; Mańko, 2021). The legal system is complex and contradictory, which gives judges the freedom to use statutes, cases, canons, maxims, principles, and authorities to back their preferred outcome.

According to Mengo (2018), the attribution of the correlation between adjudication and what judges ate for their breakfast by realists and formalists is a longstanding discourse. The judicial decision-making is often linked to frivolous factors such as what they ate with critics contending that these arguments are far-fetched while proponents cite experiments conducted by DLA. The existing literature affirms that there is evidence to an asset that judges make a decision based on extraneous factors as their repeated ruling have an element that favors the status quo, they base their rulings on precedence, which is often contested, and influenced by what judges ate ( Menga, 2018). For instance, the proponent of realist theory contends that the judges do make decisions before they back their ruling with the law, facts, and legal rules. It means that personal preferences, policy perspectives, and digestion plays a role in their decision-making process. The parties in the court proceeding influence the decision of a judge as weak, poor, or rich are favored by a judge based on their preference ( Mertz, 2016).

The realists describe adjudication as a process that involves judges responding to the underlying facts of a case when deciding on the case as opposed to the application of reasons and rules. The theory contends that the judges only consider the rules and law after making their decisions. Other realists note that the idiosyncrasies of the personality of judges and extraneous factors influence their decision-making process ( Mitchell, 2019; Mindus, 2021). For instance, when judges decide on a case when hungry, they are likely to favor the status quo regardless of the facts and judicial rules. The realists contend that the judicial outcome can be predicted as patterns such as the personality of a judge can help discern how they decide ( Mocan, 2020). In essence, the judges make a decision that falls into patterns, which correlate with the factual circumstances in the dispute. The approach that the judges take in response to the factual patterns is governed by the outcome of cases. The realists believe that the courts only enforce the uncodified and prevailing norms that they relate to the underlying factual circumstance. The crux of realists' position is that non-legal reasons explain the decision that judges take ( Owens, 2016). The shortcomings of realist findings are that they lack consistency in the use of a scientific approach to criticizing the traditional decision-making process in the courts. Moreover, the theory overemphasizes the submersion of rules and principles, as well as fact-finding. It creates a new form of verbal gymnastics and word enhancement. The critics of realism theory contend that realists perpetuate confrontational and violent society by describing justice as predetermined rather than based on rigorous analysis of facts and law ( Pasquale & Cashwell, 2018; Pichlak, 2020; and Priel, 2020). It provides the judges space to make a decision based on their interest, gut, hunch, ideology, or digestion. It encourages judges to act in a manner that perpetuates force, power, and suspicion. The theory contends that judges are faced with immense constraints that make it difficult for them in delivering dependable judgments ( Quintanilla, Alle & Hirt, 2017). These constraints include language, precedence, political influence, personal interest, and views of their colleagues.

## Discussion

To determine whether judges are influenced by extraneous factors such as what they take for breakfast, policy preference, their feeling, or cultural contexts, a controlled experiment is necessary. It would be helpful to conduct an experiment to test the variables so that the research question of whether law and judicial rules are the only factors that judges consider before making a ruling ( Richards, 2016). The current studies focus on digestion rather than the cognitive biases and heuristics that human beings are subjected to rather than on environmental conditions that influence the decision-making process. The study will bring to an end the debate on whether judges are influenced by extraneous factors apart from the law and legal rules when making their judgments.

The existing studies show that the realists are perceived as radicals while they term themselves as reformers who wanted to increase the predictability and certainty by explaining the real nature of the decision-making process among the judges ( Ranieri, 2019; Schultz & Kovacs, 2016; and Singh, 2018). The realists criticized the perception that judges make a decision based on legal principles and rules, which is considered logical reasoning. They contend that the decisions made by judges have no basis in law and judicial rules alone as non-legal rules and other factors are considered when making judgments. Though they believed in the rule of law, the realists based their arguments on the idiosyncrasy of the law ( Smejkalová, 2020). They sought to change and reform the judicial decision-making process by making it certain and efficient. The realism theorists sought the opportunity to reform legal education with the introduction of clinical legal education, which is available in many law schools in the US. The realists are social reformers who sought to make the law a tool for social action; however, they faced stiff objections ( Song, 2019). Realists believed that the policy objectives and the legal rules are intimate; thus, social reforms are premised on the knowledge of the elements that drive judicial decision rulings. As such, the realists advocated for an empirical approach to law, which has become a norm.

The realists believe that the realism theory is a down-to-earth and practical school of thought in touch with reality compared to the inflexible and scientific law of the theoretical model. Most realism theorists were eminent judges and practitioners with experience; thus, they were rarely personified by scientific theories ( Spence, 2016; Tejani, 2016). In satirical response to the realism theorists, the continental theorists remarked that what matters is not the prediction of the courts, but what legal rules and law pronounce. Realists argue that judges should base their decisions on explanations and predictions, and not present them as scientific theories. A good theory satisfies two principles including one that explains the large class of observation and makes definite predictions ( Teichman & Zamir, 2021). Theories that fail to make predictions should be abandoned in a rational world since failure to satisfy prediction means that it is controlled by a higher power ( Teichman & Zamir, 2021).

The difference between the legal realism and formalism theories is that realists contend that the decision-making process adopted by judges is founded on prediction, which formalists disagree with. If the realists stated that the judging cases are based on adherence to legal rules, they would agree with the formalists ( Troop, 2018). The difference between the two theories is the use of prediction, which the realists contend is an integral element in the judicial decision-making process. The realists play an integral role in creating a distinction between decision-making and justification as they established a division between written judgments and judicial opinions and the actual process of making the ruling ( Wei, 2021). They contend that judges use the facts and formal judicial rules to justify their choices, which means that formalists avoid the conclusion that judges make decisions based on their personal preference, personality, or hunch. Chief Justice Evan Hughes of the US Supreme Court admitted that judges often make a decision based on emotions with the rational part providing them with the basis for supporting their preference ( Weinshall, Sommer & Ritov, 2017). The judges that use convincing language and mechanical judgement are those swayed by dishonesty and emotions but that mask their lawlessness with meticulous justification.

There is a possibility that the manner in which a judge makes a ruling and their justification coincides, which means that the judicial reasoning and the decision-making often overlap. However, though the ruling may overlap, it does not suggest that it is a perfect pointer to the other. Some scholars believe that the distinction is an inaccurate representation of the judicial decision-making process and decisions ( Zygmunt, 2020). The proponents of critical legal studies contend that the actual reasoning of judges reflects their opinions and decision-making styles, which makes judicial opinion couched in a legalistic and formal manner that reflects their thinking. The realists contend that the functional legal system, the concept of law operates outside the court processes, rules, and regulations ( Winter, 2020). What the judges do and what the law says in books are different as they use extraneous factors when making their judgments. There are gaps between the enforceable rules and legal valid rules. In essence, how the rules in books are enforced against parties such as racial and ethnic minorities, the political power, and the underprivileged are different, which shows that the cultural environment impacts the decision of a judge ( Harris & Sen, 2018). The legal realists emphasize indeterminacy, on which the courts base their arguments and facts that provide judges with interpretative latitude. The judges use the latitude to construe the precedents and statutory provisions. The truth is that the varied interpretations are considered valid and legitimate when they are based on other legal actors rather than a reference to a normative standard ( Heumann *et al.*, 2019). As such, the realists depend on the scope of legal indeterminacy when making their arguments against formalism theory. Overall, legal formalists tend to avoid considering that judicial opinion fails to reflect the actual judicial reasoning.

My appeal is that the independence of the judiciary is protected by trusting in the judges by avoiding frivolous accusations that some findings are based on personal interests, inclination, and the ideology of judges rather than adherence to the rule of law and observing the judicial rules. Encouraging cynicism is dangerous as it causes one to consider their talents in defeating arguments supported by law. Reversing the trend is essential as calling out the actions of those meddling with the appointment and operations of judges will force them to rethink their decisions. Besides commenting on the development of the law, arguing critical cases, helping in the appointment of judges, making government policy, and writing legislation are imperative in reversing the trend where people attack the judges and their reasoning. Commentators should avoid criticizing the self-indulgence of the judiciary as it encourages future generations to disregard the court and constitutionalism. Conversely, judicial officers should ensure that they hold their position morally and legally defensible.

## Conclusion

The old trope that judges' decisions are based on what they ate as opposed to the law and judicial rules is a contentious discussion. The discussion is centered on whether the judges are influenced by extraneous factors other than the law and judicial rules, which the realists affirm and vehemently denied by the formalists. For formalists, the judicial decision-making process is bound by the law and judicial rules, which are perceived by realists as mechanical and irrational. Though the formalists don't agree with the extreme views of the judicial decision-making process, they nonetheless consider formal rules imperative. On the other hand, the realists contend that the judicial rules are influenced by other factors and that the law and facts are supplementary. There is a consensus among the realists that the rules and law play some role, however, the other extraneous factors are more important. While making a judgment, the judge only considers the laws and rules to support their ruling. Also, for any position, the judges find a legal basis to support their ruling using the formal rules, law, and facts. Though legal realists are perceived as radicals that advocated for unscientific decision-making procedures for judges, they raised attention to the driving force of the judicial decision-making process and factors that influence their rulings.

My submission is that constitution was created to ensure that everyone feels protected regardless of the persuasion, leaning, instinct, and beliefs of a judge. The principles are designed to help judges make rational and fair judgments; thus, their conduct should generate public trust in the judiciary. I believe that the judges make their determination based on the judicial rules and law as opposed to the political agenda, interests, and attitudes. Lack of trust in the judges can cause points of dissatisfaction when they make unpopular decisions. It brings an impetus for changes that are far-reaching that can impact the lives of people. As such, vilifying judges based on how they voted or made their judgments citing frivolous issues such as their political stand, leaning, ideology, or what they ate can cause harm to the rule of law. For instance, I consider it wrong to throw judges out of office on political grounds rather than their integrity or qualification. The hiring of Chief Justice, Supreme Court judges, and judges in federal courts receive the attention of political players, which is inappropriate since it can affect the lives of people. Installing judges based on their political inclination and ideology can impact the rulings that guide the societal fabric. It offers an opportunity for political players to fiddle with the operations and jurisdiction of the federal courts. When and if the trend continues, it will impact the judicial review that can be limited in scope, affect the removal process, or circumscribe the appointment of judges. Consequently, the judiciary will lose its independence or it will be undermined.

## Data Availability

No data are associated with this article.
