# Supplementary material for: Judicial breakfast as an external factor in judicial decision making in courts
Source: F1000Res. 2023 Jan 4;12:9. [Version 1] doi: 10.12688/f1000research.126482.1 (PMC9999048; doi:10.12688/f1000research.126482.1)
Supplement: Peer review history

# Peer review: Reviewer response for version 1

**DOI:** 10.5256/f1000research.138899.r158971

**Published:** 2023-03-09

**Authors:** Aneesh V. Pillai

**Affiliations:** 1 School of Legal Studies, Cochin University of Science and Technology, Kochi, Kerala, India

The theme of this article is an enquiry about the judicial decision-making process and the factors influencing it. The article proceeds by discussing two well-known theories such as formalism and legal realism. The theory of formalism believes that, the judicial making process always depends upon the facts and circumstances and the application of established legal principles. However, the theory of realism believes that the judicial decision-making process is largely influenced by extra-legal factors. To analyse further, the Article discusses the theories such as Danziger, Levav and Avnaim-Pesso Theory; Hunch Theory; and Democratic Theory. To substantiate the point, the authors have reviewed several kinds of literature on this topic and taken ideas from such literature.

 From the analysis, the authors identify that, when the judicial decision-making process is influenced by extraneous factors, there arises a gap between the enforcement of rules and principles. This may adversely affect the protection of interests of racial and ethnic minorities and the underprivileged. The Article identifies that, ‘there is a consensus among the realists that the rules and law play some role; however, the other extraneous factors are more important’. Thus it can be seen that, while making a judgment, the judges give predominant consideration to legal rules and principles; however, other extraneous factors may influence the mind of judges if there arises a situation of personal discretion. Thus the judgements will always have a legal base and is also influenced by judges’ character, social background and other factors. The article concludes by submitting that, fairness in judicial decision-making is an utmost necessity. Hence, the influence of extra-legal factors should be avoided.

 This article is well written in accordance with the existing standards and legal literatures. It has dealt with the issue of judicial decision making in an elaborate manner. The article cites several relevant references and incorporates the ides from such literatures. Since it has fairly covered all the relevant issues, it can be indexed without any further addition or modification’s.

Is the topic of the opinion article discussed accurately in the context of the current literature?

Yes

Are arguments sufficiently supported by evidence from the published literature?

Yes

Are all factual statements correct and adequately supported by citations?

Yes

Are the conclusions drawn balanced and justified on the basis of the presented arguments?

Yes

Reviewer Expertise:

Emerging area in International Law; Human Rights; Cyber Law; Consumer Law and Judicial Porcess

I confirm that I have read this submission and believe that I have an appropriate level of expertise to confirm that it is of an acceptable scientific standard.

# Peer review: Reviewer response for version 1

**DOI:** 10.5256/f1000research.138899.r158972

**Published:** 2023-01-26

**Authors:** Favio Farinella

**Affiliations:** 1 Center for Research in International Law and Regional Integration, National University of Mar del Plata, Mar del Plata, Buenos Aires, Argentina

The purpose of this article is to investigate the way in which judicial decisions are constructed. In this regard, the authors present the two major existing theories: legal realism and dogmatic legal interpretation. The first minimizes the normative and prescriptive element of the law and maximizes the empirical and descriptive factors. On the contrary, the dogmatic school is a method of study and legal research and its object of investigation is the norm.

 The text under analysis seeks to investigate whether judicial decisions are the product of the application of the law on the facts (dogmatic school) or if, on the contrary, other personal and social factors intervene (legal realism school). And in any case, the text aims to demonstrate if such factors exist at the time of the judicial decision, and their real incidence on the judicial decisions.

 The authors carry out a complete review of the existing doctrine and theories in this regard. Finally, in the conclusion, they offer a well-founded opinion, explaining that although there may be extra-legal factors, the judges mainly make their determination based on the judicial norms and the law without being greatly affected by the political agenda, the interests of the parties to the conflict and other attitudes of third parties.

Is the topic of the opinion article discussed accurately in the context of the current literature?

Yes

Are arguments sufficiently supported by evidence from the published literature?

Yes

Are all factual statements correct and adequately supported by citations?

Yes

Are the conclusions drawn balanced and justified on the basis of the presented arguments?

Yes

Reviewer Expertise:

International law; human rights; artificial intelligence and the law.

I confirm that I have read this submission and believe that I have an appropriate level of expertise to confirm that it is of an acceptable scientific standard.
